# Acute effects of ketamine on the pregenual anterior cingulate: linking spontaneous activation, functional connectivity, and glutamate metabolism

**DOI:** 10.1007/s00406-021-01377-2

**Published:** 2022-01-12

**Authors:** Matti Gärtner, Anne Weigand, Milan Scheidegger, Mick Lehmann, Patrik O. Wyss, Andreas Wunder, Anke Henning, Simone Grimm

**Affiliations:** 1 MSB Medical School Berlin, Rüdesheimer Straße 50, 14197 Berlin, Germany; 2 Department of Psychiatry and Psychotherapy, Charité, Universitätsmedizin Berlin, Corporate Member of Freie Universität Berlin and Humboldt-Universität Zu Berlin, Hindenburgdamm 30, 12203 Berlin, Germany; 3 Department of Psychiatry, Psychotherapy and Psychosomatics, University Hospital of Psychiatry, University of Zurich, Zurich, Switzerland; 4 Department of Radiology, Swiss Paraplegic Centre, Nottwil, Switzerland; 5 Translational Medicine and Clinical Pharmacology, Boehringer Ingelheim Pharma GmbH & Co. KG, 88397 Biberach an der Riss, Germany; 6 Advanced Imaging Research Center, UT Southwestern Medical Center, Dallas, TX USA

**Keywords:** Ketamine, Glutamate, Resting state fMRI, MR Spectroscopy

## Abstract

Ketamine exerts its rapid antidepressant effects via modulation of the glutamatergic system. While numerous imaging studies have investigated the effects of ketamine on a functional macroscopic brain level, it remains unclear how altered glutamate metabolism and changes in brain function are linked. To shed light on this topic we here conducted a multimodal imaging study in healthy volunteers (*N* = 23) using resting state fMRI and proton (^1^H) magnetic resonance spectroscopy (MRS) to investigate linkage between metabolic and functional brain changes induced by ketamine. Subjects were investigated before and during an intravenous ketamine infusion. The MRS voxel was placed in the pregenual anterior cingulate cortex (pgACC), as this region has been repeatedly shown to be involved in ketamine’s effects. Our results showed functional connectivity changes from the pgACC to the right frontal pole and anterior mid cingulate cortex (aMCC). Absolute glutamate and glutamine concentrations in the pgACC did not differ significantly from baseline. However, we found that stronger pgACC activation during ketamine was linked to lower glutamine concentration in this region. Furthermore, reduced functional connectivity between pgACC and aMCC was related to increased pgACC activation and reduced glutamine. Our results thereby demonstrate how multimodal investigations in a single brain region could help to advance our understanding of the association between metabolic and functional changes.

## Introduction

Over the last 2 decades, ketamine has become an important research tool to investigate rapid antidepressant mechanisms of action [1]. It has been repeatedly shown that antidepressant effects of ketamine occur within 24 h after a single dose [2]. On the one hand, this allows for the study of antidepressant mechanisms, as it enables to establish a direct link between the drug and its effects. But on the other, the molecular and systemic changes ketamine induces in the central nervous system are notoriously complex and it is a matter of ongoing research to pin down which of these changes bring forward the antidepressant effects. Despite many unanswered questions, there is a broad consensus that the antidepressant effects of ketamine are linked to changes in the glutamatergic system [3], and there is evidence that altered glutamatergic signaling leads to intercellular signaling cascades that induce synaptic plasticity, which has been associated with ketamine’s antidepressant effects in behavioral animal models [4]. Furthermore, a considerable amount of imaging studies investigated the effects of ketamine on functional brain activations and networks in humans [5]. Although the results of this research have not yet converged, and no clear antidepressant mechanism of action has been identified, some interesting themes and involved brain regions, such as the anterior cingulate cortex [6], have emerged. However, evidence remains sparse how ketamine-induced molecular changes in the glutamatergic system are linked to macroscopic functional activation and network changes in the human brain.

Considering the important role of glutamate (Glu) in synaptic transmission and plasticity, perturbation of the glutamatergic system is considered one of the factors involved in the pathophysiology of depression, while Glu modulation may induce rapid relief of depressive symptoms [7–9]. Glutamate is synthesized in the presynaptic terminal from glutamine (Gln), and then released into the synaptic cleft. Astrocytes provide the primary mechanism for clearance from the synaptic cleft, by means of uptake by amino acid transporters. Once in the astrocyte, Glu is converted to Gln by the Gln synthetase. Gln is then released into the extracellular space for uptake into the excitatory and the inhibitory neurons, where it is converted back to Glu via glutaminase. This is referred to as the glutamate/glutamine cycle [10]. Astrocytic pathology and subsequent alterations in this cycle have been implicated in the physiology of depression [9, 11] with astrocyte-related reductions in conversion of Glu to Gln leading to downstream Glu reduction [12]. Proton (^1^H) magnetic resonance spectroscopy (MRS) is the only non-invasive method able to directly measure glutamate and glutamine levels in vivo. In some MRS studies, elevated Gln and Glu levels have been linked to depression, while other studies reported reduced concentrations of both Gln and Glu [13–18]. As it is difficult to separate Glu clearly from its precursor and metabolite, Gln, the two compounds are often measured together as Glx and accordingly, inconsistent alterations in Glx have also been reported in depression [19–23]. Thus, based on the current stage of research, it remains unclear how exactly perturbation of the glutamatergic system and depression are linked. A more detailed understanding of this relationship would be helpful to pin down the antidepressant mechanisms of rapid-acting glutamatergic antidepressants such as ketamine. Furthermore, baseline metabolite levels in depressed patients could be established as an indicator of treatment response to medication targeting glutamatergic receptors [24].

Several lines of investigation have shown that ketamine increases prefrontal Glu levels through NMDA receptor inhibition and subsequent AMPA receptor activation [25–27]. Interestingly, the pregenual anterior cingulate cortex (pgACC) has been found to exhibit above average AMPA receptors and below average NMDA receptor densities (compared with whole cingulate cortex) [28] and regional variations of Glu and Gln concentrations have been shown to follow these receptor fingerprints [29]. Abdallah et al. [30] reported that ketamine increases prefrontal glutamate–glutamine cycling, thereby providing the most direct evidence in humans that ketamine increases glutamate release in the prefrontal cortex. Changes in the pgACC occur within the first 30 min after the start of the ketamine infusion, which supports the idea that the Glu burst happens quite early [31]. Accordingly, several 1H-MRS investigations noted increased glutamatergic metabolite levels in healthy volunteers [25, 26] and increased Glx levels in depressed subjects [31] during acute ketamine administration; the ratio of Gln to Glu was also found to be increased 24 h after acute ketamine administration in healthy volunteers [32]. Nevertheless, other investigations found no significant changes in Glu measurements 1 h post-ketamine infusion in healthy volunteers [33], or three and 48 h later in depressed subjects [34]; variations in voxel location, timing of the scan, imaging parameters, and sample size may explain these discrepant findings. Evans et al. [35] reported that ketamine did not affect Glu or Gln levels in the pgACC in depressed subjects 24 h post-ketamine infusion and that antidepressant response was not predicted by baseline levels. On the other hand, there are reports of a prediction of treatment response by baseline levels of glutamatergic metabolites as well as of an association between lower Glx response to acute ketamine administration and antidepressant response [36, 37].

As of today, a relatively large number of imaging studies have investigated the effects of ketamine in the human brain [5]. Although results do not converge into a clear pattern of effects yet, several findings have been replicated in independent studies. The anterior cingulate cortex (ACC), and especially the pre- and subgenual subdivisions, have been repeatedly implicated in the effects of ketamine [6]. The pregenual ACC (pgACC) plays an important role in emotional processing and establishing mood states [38, 39], and abnormal pgACC activity has been implicated in depressive pathology [40, 41]. Furthermore, ketamine-induced changes in functional connectivity between the right lateral PFC and the sgACC have been linked to the reduction of depressive symptoms [42], which could indicate restored prefrontal cognitive control over emotional processing. Thus, it is likely that ketamine-induced alterations in pgACC and sgACC activity are linked to relief of depressive symptoms. However, despite the extensive study of ketamine using brain imaging in healthy and depressed human subjects, the exact antidepressant mechanism of ketamine remains unclear. Investigating the relationship between ketamine induced metabolic and functional brain changes via multimodal brain imaging is a promising approach to advance our understanding of how ketamine exerts its rapid antidepressant effects.

To the best of our knowledge, the link between ketamine-induced alterations in glutamatergic signaling, and altered functional activation, and connectivity has only been investigated in two previous studies. One of these, however, focused on changes 1 h and 24 h after ketamine administration [43] and reported that altered Gln/Glu concentration 24 h after ketamine was associated with increased functional connectivity within the default mode network (DMN). The study by Kraguljac et al. [44] reported neurometabolite levels and functional connectivity during ketamine infusion, however, focused on the hippocampus which is not part of the neural circuitry of depression and furthermore did not distinguish between Glu and Gln.

Based on prior work demonstrating that changes in glutamatergic neurotransmission in pgACC, a region implicated both in the effects of ketamine and in emotional processing, occur early after the start of the ketamine infusion, our study aimed to investigate acute effects of ketamine on pgACC spontaneous activity, functional connectivity, and Gln/Glu metabolite concentration using a multimodal imaging approach.

## Methods

### Participants

The study was performed at the University of Zurich by the Department for Psychiatry, Psychotherapy and Psychosomatics (Psychiatry University Hospital Zurich) and the Institute for Biomedical Engineering (ETH Zürich). Twenty-three healthy subjects (*n* = 23, mean age, 25.5; 12 males) participated in the MRI study. Resting state fMRI data were available for the entire sample (*N* = 23). Complete MRS data were available for a sub-sample of *N* = 16 participants. MRS data of five subjects had to be excluded because of an error in the MRS sequence. Additionally, MRS data of two subjects could not be analyzed because of a missing high-resolution 3D T1 weighted sequence, which was needed for tissue signal correction. All subjects underwent a medical examination and psychiatric interview based on the Brief Psychiatric Rating Scale (BPRS; [45]) and the Hamilton Rating Scale for Depression (HAMD; [46]). Only medication-free subjects that were healthy according to the physical examination, electrocardiogram, and blood and urine analyses were included in the study. Exclusion criteria were a history of psychiatric/neurological diseases, drug abuse, concurrent medication, cardiovascular disease, anemia, thyroid disease, any somatic disease affecting drug metabolism and excretion (e.g., renal or liver disease), MR exclusion criteria, pregnancy and left-handedness.

### Study design

Subjects completed two separate MRI sessions on a Philips Achieva 3T whole-body MR unit. The first session served as physiological baseline and subjects were not infused with any (pharmacological) agent. In the second session, S-ketamine (Ketanest, Pfizer, Zurich, Switzerland) was administered as an intravenous bolus of 0.12 mg/kg approximately 25 min prior to the MRI, followed by a continuous infusion of 0.25 mg/kg/h during the entire MR scanning period. During the acquisition of resting state, subjects were told to lie still in the scanner with their eyes closed. The functional images were collected in 10 min runs (200 volumes). The interval between the two sessions was 3 days on average.

### Data acquisition and analyses

#### Resting state fMRI

The functional images were acquired with a sensitivity-encoded single-shot echo-planar imaging sequence (SENSE-sshEPI) [47] in 10 min runs (200 vol). Following acquisition parameters were used: TE = 35 ms; field of view (FOV) = 22 cm, acquisition matrix = 80 × 80 interpolated to 128 × 128, TR 3000 ms, flip angle 82°, voxel size = 2.75 × 2.75 × 4 mm, 32 contiguous axial slices (placed along the anterior–posterior commissure plane) and sensitivity-encoded acceleration factor R = 2.0).

Three-dimensional T1-weighted anatomical scans were obtained for structural reference for both, placebo and ketamine conditions individually (T1 3D FFE sequence: TR/TE = 9.3/4.6 ms, flip angle = 8°, 160 sagittal slices, FOV 240 × 240 mm, voxel size 1 × 1 × 1 mm).

Resting state fMRI data were analyzed in MATLAB (VersionR2018b) using SPM12 and the CONN toolbox (Version 20b; [48]). Preprocessing of fMRI data included removal of the first five scans, motion correction (realignment and unwarping), slice-timing correction, automatic detection of artifactual scans (ART-based scrubbing; 97th percentile), normalization to MNI space (structural target resolution: 1 mm, function target resolution: 2 mm), and spatial smoothing (8 mm). During the denoising step in CONN single-subject linear regression analyses were performed to remove the effects of head motion (12 total motion covariates: 6 motion parameters plus temporal derivatives), physiological artifacts (10 total CompCor eigenvariates: 5 each from eroded WM and CSF masks), and artifactual scans. The resulting residual BOLD time series were band-pass filtered (0.01–0.1 Hz). Spontaneous neural activity was calculated using *fractional amplitude of low frequency fluctuations* (fALFF) as implemented in the CONN toolbox [49]. The pgACC ROI used for the functional imaging analysis was created based on previous publications (MNI coordinates: 0, 42, 2; diameter: 10 mm; [50, 51]).

#### MRS

The high-resolution 3D T1 was used to plan the MRS acquisition and calculate the tissue composition of the voxel. Spectra were acquired with a maximum echo-sampled J-resolved PRESS sequence (JPRESS; [52]) including second-order B0 shimming with VAPOR water suppression [53] and outer volume suppression (OVS) and inner volume saturation (IVS) [54, 55]. The voxel size was 25 × 18 × 20 mm^3^ (AP × LR × FH). JPRESS was implemented as two-dimensional echo time series with a minimum echo time (TE) of 30 ms and repetition time (TR) of 1600 ms. 100 echo steps (step size = 2 ms) with 8 water suppressed signal averages (NSA) per echo step and non-water suppressed reference were acquired covering the range from 30 to 228 ms with a total number of signal averages of 800 (acquisition time: 24 min). The 2D technique allows disentangling of the signals of overlaying metabolite resonances by encoding the chemical shift information in the first dimension and the J-coupling information in the second dimension. Therefore, the signals of usually overlapping (in 1D) metabolites such as Glutamine (Gln) and Glutamate (Glu) can be identified independently.

**Fig. 1:**
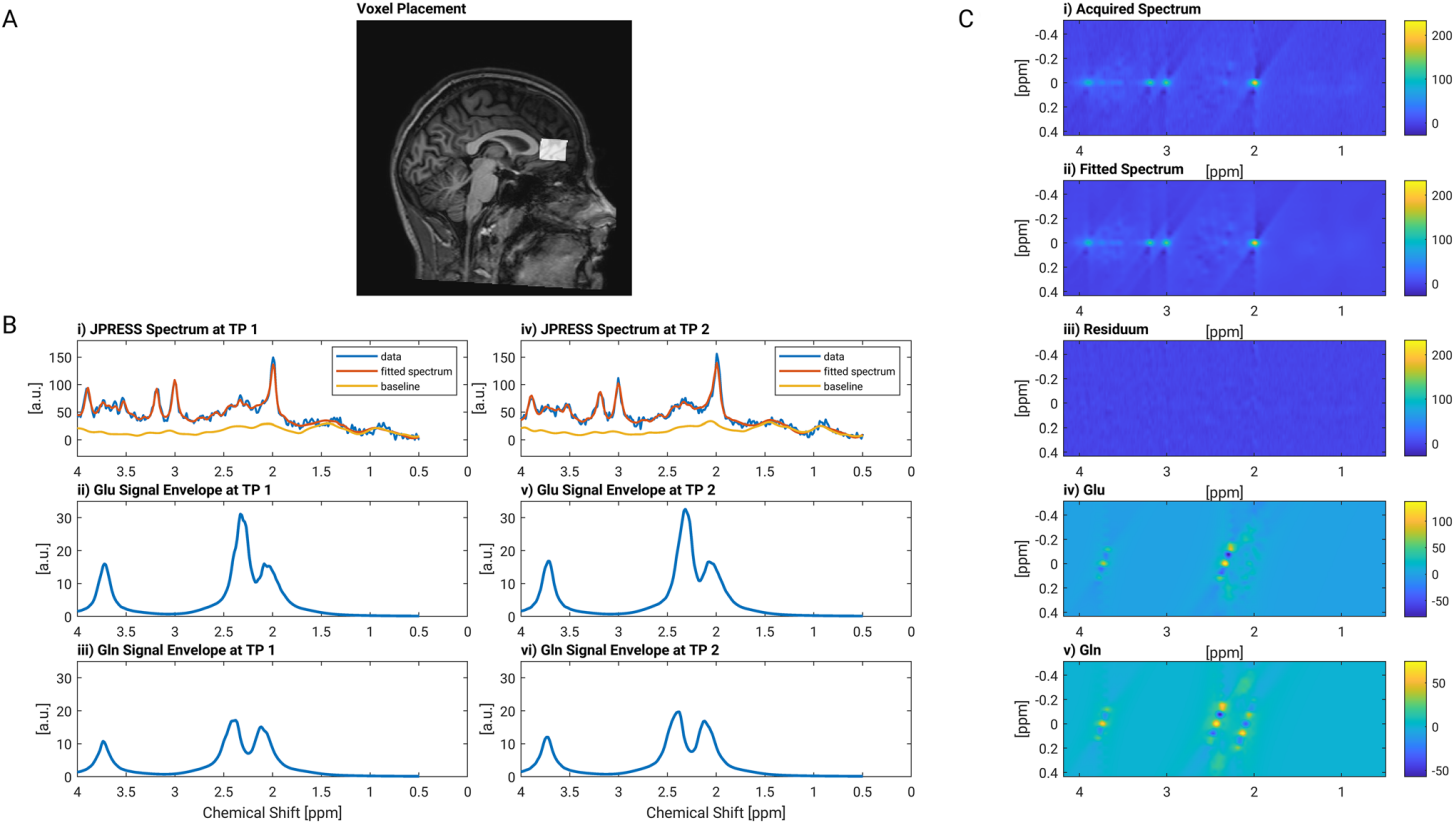
MRS analyses. **A** Representative spectroscopy voxel placement in the pregenual anterior cingulate cortex (sagittal view). **B** Representative one-dimensional projections of the 2D JPRESS data: The measured data (blue), the fitted spectrum (red) and the baseline (orange) are shown for the first (left rows; (i)] and second timepoint (right rows, (iv)]. In addition, the individual signal contribution envelope of Glutamate (ii) and (v) and Glutamine (iii) and (vi) is shown at the first and second time point. **C** Representative two-dimensional JPRESS data: The acquired spectrum (i), the fitted spectrum (ii) and the fit error (residuum, iii) are shown including the signal contributions of glutamate (iv) and glutamine (v). The common and distinct frequency pattern is shown to help disentangle the signal from Glu and Gln

The MRS data were analyzed with ProFit2 (Prior-knowledge Fitting) including 18 metabolites [56]. The basis set consisted of 18 metabolites including Glu, Gln and GABA. A chemical shift range was covered in the first dimension from 0.5 to 4 ppm and in the second dimension from − 0.4 to 0.4. Processing steps included a Hankel singular value decomposition (HSVD) water filter, eddy current, and zero- and first-phase correction. Artefact inspection for bad water suppression, line shape distortion or ghosting was conducted visually. The amount of water signal measured in the non-water suppressed reference acquisition was used as internal reference. In addition, with a custom-written MATLAB script tissue composition correction (CSF, WM, GM) was conducted to report metabolite concentration values with respect to internal water [57]. Results from the MRS analysis for one representative subject are shown in Fig. 1.

### Statistical analysis

For the whole-brain fMRI analyses the results were considered significant at the voxel-level *p* < 0.001, at the whole-brain level, cluster-corrected at p-FDR < 0.05. For some exploratory analysis and visualization purposes, we used a voxel threshold of *p* < 0.05. This liberal voxel threshold was penalized with a strict cluster correction level of p-FDR < 0.001. Correlations between imaging modalities were calculated using Pearson’s correlation coefficient (alpha level = 0.05). For each modality, combination results were FDR corrected. However, uncorrected results are reported as well, and marked as such.

## Results

### Spontaneous brain activation

**Fig. 2:**
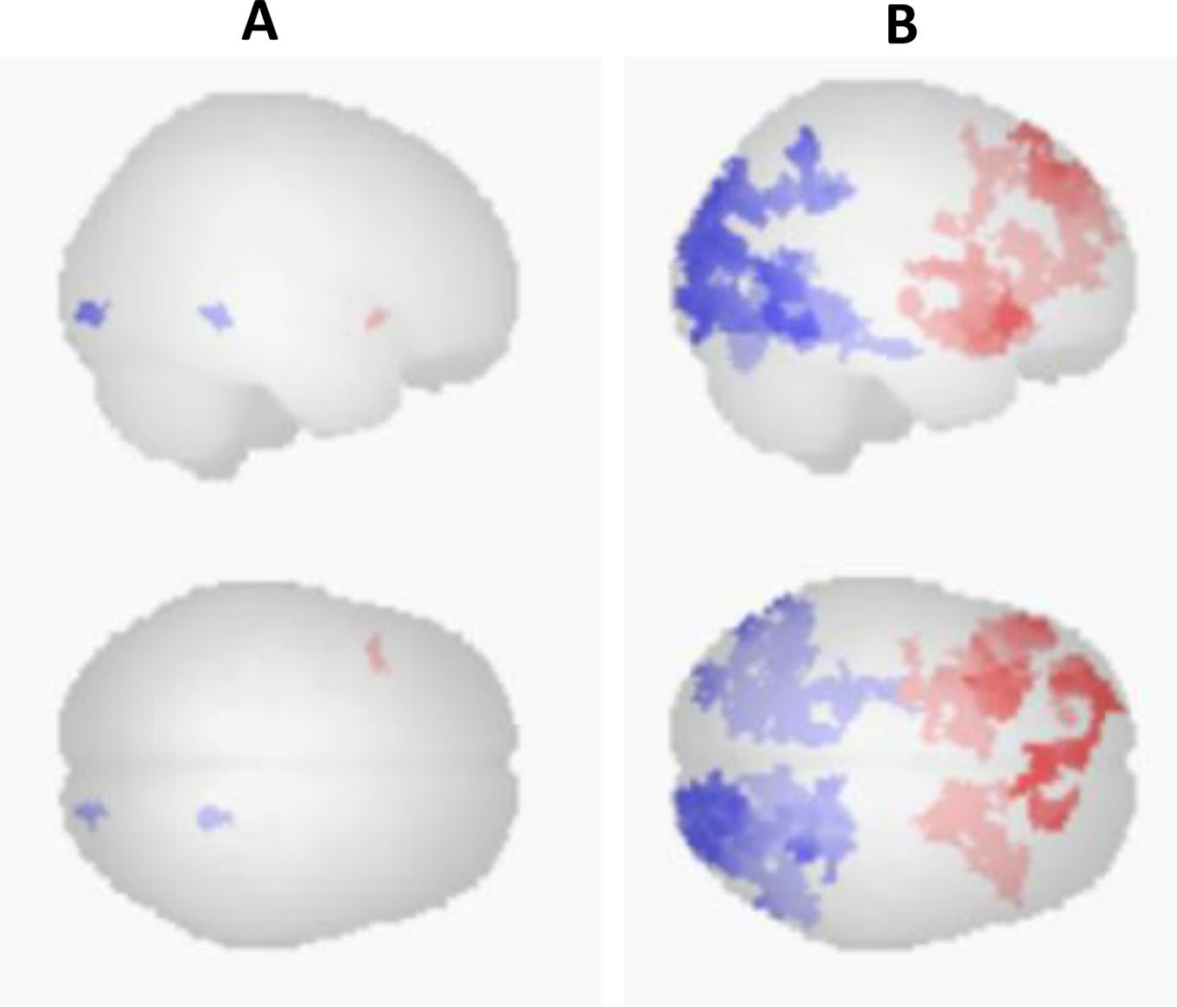
Spontaneous brain activation during ketamine. Increased activation is shown in red color, decreased activation is shown in blue color. **A** Activation changes with standard settings for cluster-based inference. **B** Activation changes with exploratory settings for cluster-based inference

The whole-brain analysis of spontaneous brain activation revealed stronger activation of the left anterior insula, and deactivation in the right lingual gyrus and the right occipital pole under ketamine (Fig. 2A, Table 1). The exploratory analysis revealed a clear picture of activation in multiple prefrontal brain regions, and deactivation in multiple posterior brain regions (Fig. 2B). The ROI analysis in the pgACC revealed no significant change between baseline activations and activations during the ketamine infusion (*t*(22) = − 1.38, *p* = 0.18). On the descriptive level, pgACC activations were increased during ketamine (*M* = 0.13, SD = 0.51) compared to baseline (*M* = − 0.05, SD = 0.51).

**Table 1:** Cluster statistics for significant spontaneous brain activation during ketamine

Cluster (x,y,z)	Region	Size	p-FDR	p-uncorrected
+ 20 − 44 − 06	Lingual gyrus right	55	0.002	0.00002
+ 18 − 96 − 06	Occipital pole right	40	0.009	0.00015
− 44 + 16 − 08	Insular cortex left	33	0.017	0.00043

### Resting state functional connectivity

**Fig. 3:**
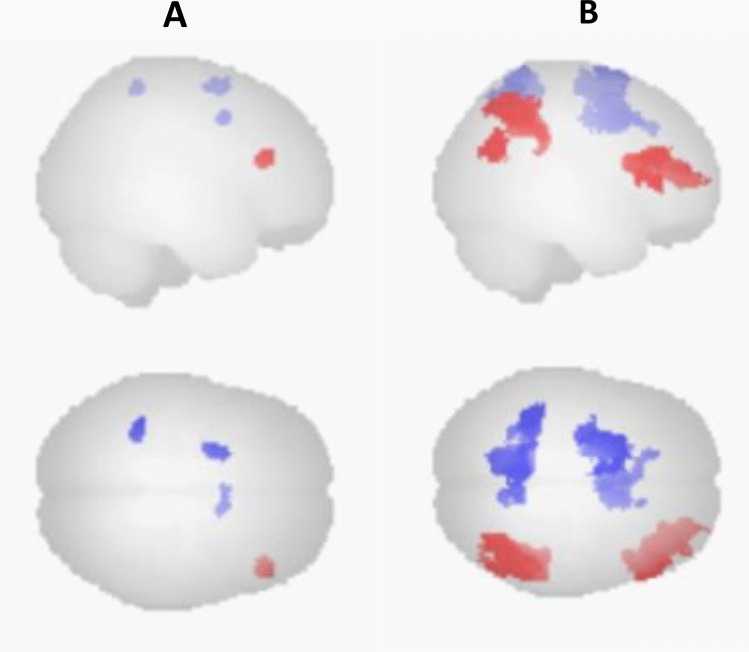
Altered resting state functional connectivity during ketamine of the pgACC seed region. Increased activation is shown in red color, decreased activation is shown in blue color. **A** Activation changes with standard settings for cluster-based inference. **B** Activation changes with exploratory settings for cluster-based inference

The analysis of resting state functional connectivity (rsFC) changes during ketamine revealed increased rsFC from the pgACC seed to the right frontal pole, and decreased rsFC to the anterior mid cingulate cortex (aMCC), the left superior frontal gyrus, and the left superior parietal lobe, (Fig. 3, Table 2).

**Table 2:** Cluster statistics for significant rsFC changes of the pgACC during ketamine

Cluster (x,y,z)	Region	Size	p-FDR	p-uncorrected
+ 48 + 34 + 20	Frontal pole right	102	0.008	0.0004
− 22 + 06 + 62	Superior frontal gyrus left	100	0.008	0.0004
− 34 − 46 + 62	Superior parietal lobule left	75	0.020	0.0016
+ 14 + 02 + 42	Anterior mid cingulate cortex	68	0.022	0.0024

### Metabolite concentration

The analysis of glutamate signaling in the pgACC revealed no significant changes for Glu, Gln, and the Gln/Glu ratio. However, a trend towards increased Glu concentration during ketamine was observed. On the descriptive level, metabolite concentrations were increased for Glu and decreased for Gln and the Gln/Glu ratio (Table 3).

**Table 3:** Metabolite concentrations at baseline and during ketamine

Metabolite	Baseline	Ketamine	*T* statistic	*P* value
Glu	4.82 (0.78)	5.24 (0.89)	− 1.98	0.066
Gln	1.25 (0.52)	1.14 (0.50)	0.84	0.42
Gln/Glu	0.26 (0.09)	0.22 (0.08)	1.64	0.12

### Activity changes in pgACC linked to altered metabolite concentration

**Fig. 4:**
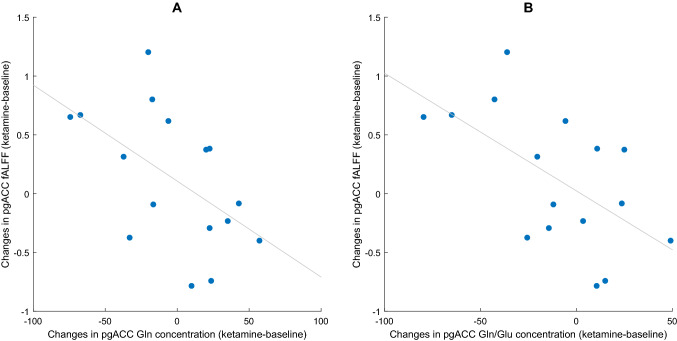
Relationship between changes in pgACC activation and metabolite levels. **A** changes in Gln levels and **B** changes in Gln/Glu levels

Correlation analyses (Pearson’s correlation coefficient) between pgACC activity changes and changes in metabolite concentration showed a significant association for Gln (*r* = − 0.54, *p* = 0.033) and the Gln/Glu ratio (*r* = − 0.59, *p* = 0.017). Increased activity in the pgACC during ketamine was linked to lower Gln concentration in the pgACC and to a lower Gln/Glu ratio (Fig. 4). No significant relationship was observed for Glu (*r* = 0.17, *p* = 0.52).

### FC changes of the pgACC linked to altered metabolite concentration

**Fig. 5:**
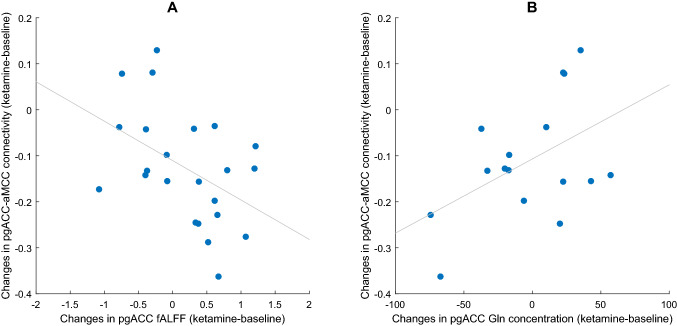
Relationship between changes in pgACC functional connectivity, activation, and metabolite levels. **A** Relationship between pgACC activation and pgACC-aMCC rsFC. **B** Relationship between Gln changes and pgACC-aMCC rsFC

Exploratory correlation analyses were carried out with the four clusters that showed rsFC changes to the pgACC seed region (cp. Table 2). It was tested whether these rsFC changes were linked to changes in pgACC activity, or changes in metabolite concentration. A relationship was observed between pgACC activation and reduced rsFC to the aMCC (*r* = − 0.45, *p* = 0.032, Fig. 5A). Furthermore, a marginally significant relationship was observed between Gln levels and pgACC-aMCC connectivity (*r* = 0.48, *p* = 0.062, Fig. 5B).

## Discussion

In this multimodal imaging study, we investigated acute effects of ketamine on spontaneous brain activation, functional connectivity, and metabolism in the glutamatergic system. In particular, we investigated how changes in the individual imaging modalities were interrelated to gain a better understanding of how molecular and macroscopic brain changes induced by ketamine are linked. We focused our analysis on the pregenual anterior cingulate cortex (pgACC) because ketamine-induced brain changes have been repeatedly reported in this region, and the specific receptor profile of this region suggests that the antidepressant mechanism of ketamine might be linked to metabolic changes in this region.

ROI analysis in the pgACC showed no significant change between baseline activation and activation during the ketamine infusion. While previous reports are rather consistent regarding decreased activation in the sgACC during a ketamine infusion [58–60], evidence for pgACC is less clear [6]. Downey et al. [61] found increased activation during ketamine in a region at the border between the pgACC and the sgACC, and Javitt et al. [62] found widespread frontal activation that included the pgACC during ketamine. These findings suggest that ketamine-induced activity changes in the pgACC and sgACC could point into opposite directions. Further, regarding spontaneous brain activations during ketamine, the whole-brain analysis conducted in our study showed increases in the left anterior insula and decreases in occipital regions. Ketamine-induced activation of the insula has also been previously reported in healthy subjects [60, 63]. The insula is a central node of the salience network (SN) and thought to mediate interpretation of sensory information that contributes to emotional states [64]. Furthermore, strong connectivity to nodes of the anterior DMN underlines the insula’s hypothesized role in switching between the DMN and executive control network (ECN; [65, 66]). Thus, increased activation of the insula during ketamine might be linked to switching from DMN to activation of other networks. This is line with previous findings suggesting that the antidepressant effects of ketamine are linked to reduced activity in brain networks related to self-referential processing [5]. Interestingly, a noticeably clear pattern of frontal activation, and posterior deactivation was observed when a more liberal statistical voxel threshold was applied. Strong frontal activation patterns induced by ketamine correspond to “hyperfrontality” reported in several previous studies and might be related to altered interpretation of visual and spatial information about the external and internal reality [67–70].

Stronger functional connectivity (FC) during ketamine was observed between the pgACC and the right frontal pole. FC between the right frontal pole and the subgenual ACC has previously been linked to the antidepressant effects of ketamine 24 h after the infusion [42] and might reflect the restoration of cognitive control over aberrant emotion processing in limbic structures [5, 71]. Decreased FC was observed between the pgACC and the anterior mid cingulate cortex (aMCC), a region implicated in cognitive aspects of motor control [72], and the experience of negative affect and pain [73]. As the pgACC is involved in the subjective experience of affect [6], its decoupling from the aMCC might be linked to altered processing of emotion. The decoupling from a region involved in motor control might also be linked to the feeling of disembodiment, which is a core feature of the subjective experience during a ketamine infusion [74].

No absolute changes in metabolic Glu, Gln, and Gln/Glu concentrations were observed, but there was a trend towards Glu increases during ketamine and descriptively Gln and Gln/Glu were reduced during ketamine. The lack of significance could be due to the rather small sample size in our study, but it should be noted that on the descriptive level the results regarding Glu are consistent with previous findings and support the idea that changes in glutamatergic neurotransmission happen quite early after the start of the ketamine infusion [31, 60]. Increased Glu concentration after ketamine has been linked to NMDA receptor inhibition and subsequent AMPA receptor activation [25–27]. Since the pgACC exhibits above average AMPA and below average NMDA receptor densities [28], the Glu response to a single subanesthetic dose of ketamine may be associated with the histoarchitectonical receptor fingerprint of this brain region.

Regarding cross-modality associations, our results showed that increased pgACC activation was linked to lower Gln concentration and a lower Gln/Glu ratio in the pgACC. Gln is seen as the non-neuroactive precursor and metabolite in the glutamate–glutamine cycle, enabling the right amount of physiological Glu neuron firing [75]. Some studies in depressed subjects reported increased Gln levels [11, 30, 76, 77] as well as lower baseline Gln predicting better clinical outcome [78]. Along that line, a recent study by Milak et al. [36] demonstrated that the lower the Glx response to ketamine, the better the antidepressant response. Activity in pgACC during emotional and cognitive tasks not only predicts antidepressant response to ketamine [79, 80], but the pgACC is currently the best supported candidate for a general neuroimaging biomarker for antidepressant response [81]. It has been proposed that an increased activity state of the pgACC may represent its treatment-responsive mode and be specifically important for clinical effects of rapid-acting glutamatergic drugs such as ketamine [61, 81, 82]. Therefore, our findings might indicate that acute ketamine administration increases pgACC activity, which would correspond to a treatment-responsive mode in depressed subjects. This might be accompanied by a surge of Glu, but initially lower Glu conversion in glial cells, resulting in a decreased Gln concentration as well as a lower Gln/Glu ratio [16]. Though speculative, in depressed patients, these changes might then eventually trigger the antidepressant effects of ketamine that occur within the first hours after a single dose of ketamine. Findings by Li et al. [32] indicate a reversal of this pattern 24 h after ketamine, i.e., an increased Gln/ Glu ratio in the pgACC, which might reflect secondary effects. To our knowledge, this is the first study reporting linkage between ketamine-induced changes in glutamatergic metabolites and spontaneous activation in the same region. While Li et al. [43] reported an association of glutamatergic metabolites and FC within the DMN as well as between FC and spontaneous brain activation at 1 h and 24 h after ketamine, they did not investigate the acute stage and did not report on all cross-modality associations.

Several other studies have reported positive associations, BOLD responses and glutamatergic metabolite concentrations [83, 84]; however, a recent systematic review reports no coherent relationship between glutamate and BOLD activation [83]. One possible reason for this could be that the majority of studies did not separate Glu and Gln due to methodological difficulties, but rather report the two compounds together as Glx. Our finding points out the importance of detailed investigations of both compounds to better understand the effects of ketamine on the glutamatergic system, since Glx measurement might obscure important findings that are linked to only one of the metabolites.

Our results also suggest that lower Gln concentration and stronger spontaneous activation of the pgACC during ketamine might be associated with reduced functional connectivity to the aMCC. While the FC result has been discussed above, this result suggests that both altered activation and metabolism are also linked to this change in FC. It is intriguing to speculate about the directionality of the relationships in this triangle, however, due to the purely correlative nature of this analysis such inferences cannot be made. It could be hypothesized that altered glutamatergic signaling in the pgACC brings forward changes in functional activations, and connectivity (and not the other way around). To investigate this hypothesis, upcoming studies could include a condition in which glutamatergic signaling is blocked by another compound such as Lamotrigine.

The main limitation of this study is the rather small sample size, and the results reported here must be replicated in larger cohorts. However, the reported effect sizes in the correlation analyses suggest meaningful results, and the scatterplots show that effects are not driven by outliers. Furthermore, to shed some more light on ketamine’s effect on glutamatergic neurotransmission, future studies should not only include a control group, but also an experimental group where Glu signaling is inhibited.

In conclusion, our results demonstrate how multimodal investigations in a single brain region could help to advance our understanding of how metabolic changes in the glutamatergic system are linked to changes in functional activations and connectivity.
